# DFA as a window into postural dynamics supporting task performance: does choice of step size matter?

**DOI:** 10.3389/fnetp.2023.1233894

**Published:** 2023-08-07

**Authors:** Patric C. Nordbeck, Valéria Andrade, Paula L. Silva, Nikita A. Kuznetsov

**Affiliations:** ^1^ Department of Psychology, Lund University, Lund, Sweden; ^2^ Center for Cognition, Action, and Perception, Department of Psychology, University of Cincinnati, Cincinnati, OH, United States; ^3^ Department of Rehabilitation, Exercise, and Nutrition Sciences, College of Allied Health Science, University of Cincinnati, Cincinnati, OH, United States

**Keywords:** detrended fluctuation analysis, center of pressure, step size, diffusion plot, postural control, virtual reality

## Abstract

**Introduction:** Detrended Fluctuation Analysis (DFA) has been used to investigate self-similarity in center of pressure (CoP) time series. For fractional gaussian noise (fGn) signals, the analysis returns a scaling exponent, DFA-α, whose value characterizes the temporal correlations as persistent, random, or anti-persistent. In the study of postural control, DFA has revealed two time scaling regions, one at the short-term and one at the long-term scaling regions in the diffusion plots, suggesting different types of postural dynamics. Much attention has been given to the selection of minimum and maximum scales, but the choice of spacing (step size) between the window sizes at which the fluctuation function is evaluated may also affect the estimates of scaling exponents. The aim of this study is twofold. First, to determine whether DFA can reveal postural adjustments supporting performance of an upper limb task under variable demands. Second, to compare evenly-spaced DFA with two different step sizes, 0.5 and 1.0 in log_2_ units, applied to CoP time series.

**Methods:** We analyzed time series of anterior-posterior (AP) and medial-lateral (ML) CoP displacement from healthy participants performing a sequential upper limb task under variable demand.

**Results:** DFA diffusion plots revealed two scaling regions in the AP and ML CoP time series. The short-term scaling region generally showed hyper-diffusive dynamics and long-term scaling revealed mildly persistent dynamics in the ML direction and random-like dynamics in the AP direction. There was a systematic tendency for higher estimates of DFA-α and lower estimates for crossover points for the 0.5-unit step size vs. 1.0-unit size.

**Discussion:** Results provide evidence that DFA-α captures task-related differences between postural adjustments in the AP and ML directions. Results also showed that DFA-α estimates and crossover points are sensitive to step size. A step size of 0.5 led to less variable DFA-α for the long-term scaling region, higher estimation for the short-term scaling region, lower estimate for crossover points, and revealed anomalous estimates at the very short range that had implications for choice of minimum window size. We, therefore, recommend the use of 0.5 step size in evenly spaced DFAs for CoP time series similar to ours.

## 1 Introduction

Detrended Fluctuation Analysis (DFA) was introduced by Peng et al. (1994) to characterize the spatial distribution of DNA nucleotides—are they distributed randomly, or do they occur in patches? The method has also proven useful in estimating self-similarity and scaling properties in physiological and behavioral time series (Kuznetsov and Rhea, 2017; Liddy and Haddad, 2018; Ravi et al., 2020). DFA has gained popularity because it is relatively straightforward to implement and interpret (Kuznetsov and Rhea, 2017). DFA characterizes the diffusion property of signals based on the fGn/fBm model (Mandelbrot and van Ness, 1968). In this model, fBm is defined as a random process, *x*_*t*_, with variance that grows over time, *t*, as:

$$var\left(x_{t}\right)∼t^{2H}$$

Where *H* is the Hurst exponent (also see Delignières and Marmelat, 2013). The Hurst exponent determines the correlation structure of the signal and can take any real value within the interval between 0 and 1. For *H* = 0.5, fBm captures normal diffusion (or standard Brownian motion) in which variance grows proportionally to the duration of observation. *H* < 0.5 and *H* > 0.5 capture under- and hyper-diffusive Brownian processes, respectively. As the names suggest, in under-diffusive processes, variance in the measure state does not increase over time as fast as expected in normal diffusion, and, in hyper-diffusion, it increases faster than expected. For discrete signals, fGn is obtained by differencing successive fBm values. Accordingly, for an fGn process, *H* = 0.5 implies that successive increments of the fBm process are independent and identically distributed, i.e., “random noise.” *H* < 0.5 implies anti-persistent dynamics, in which consecutive increments tend to go in opposite directions from the previous ones (tendency to “correct” about the mean). *H* > 0.5 implies persistent dynamics, with successive increments moving in the same direction (Almurad and Delignières, 2016; Liddy and Haddad, 2018). Cumulative summation of an fGn process results in an fBm.

DFA quantifies the extent to which a recorded signal exhibits long-range correlations and captures how its variance (or fluctuations) grows as a function of timescales (Peng et al., 1994; 1995). Typical steps in the analysis involve integrating the time series and splitting the signal into multiple non-overlapping windows. A fluctuation function is then calculated as an averaged variance of the linearly detrended signal within each window. This process is repeated for a range of window sizes, sometimes referred to as “scales” (Almurad and Delignières, 2016; Kuznetsov and Rhea, 2017). The alpha scaling exponent, DFA-α, is then estimated as a slope of the linear regression of the fluctuation function over a range of scales on a log-log plot (or diffusion plot). The magnitude of DFA-α estimates the temporal correlations or variability structure in the signal: DFA-α ≈ 0.5 indicates no temporal correlations or statistical independence among consecutive fluctuations (i.e., white noise); DFA-α < 0.5 means negative correlations or anti-persistence. A DFA-α > 0.5 indicates long-range positive correlations or persistent structure whose characteristics differ in important ways depending on the degree of correlation. A DFA-α ≈ 1 indicates subtle positive temporal correlations assumed to be a product of a delicate mix of deterministic and stochastic processes characteristic of fractal dynamics (or pink noise). DFA-α between 1 and 1.5 indicates an under-diffusive fBm process, while DFA-α between 1.5 and 2 indicates particularly high temporal correlations characteristic of hyper-diffusive fBm processes. To convert DFA-α to *H*, the following rule is used: When the original time series is fGn-like, *α* = *H*, and when the time series is fBm-like, *H* = α−1 (Liddy and Haddad, 2018).

DFA has been extensively used in the study of postural control in humans (Duarte and Stenard, 2008; Blázquez et al., 2009; Moreno et al., 2022). The signal for DFA analysis in this context represents subtle changes or adjustments in overall body orientation (or posture) that happen even when a person is seemingly standing still. These postural adjustments can be tracked through sequential measures of the location of the body’s center of pressure (CoP)—the central (average) point of bodily forces acting on the ground in two directions (Anterior-Posterior [AP] and Medial-Lateral [ML]). In this context, DFA has been employed to capture the degree and type of dependencies observed in CoP displacements (or fluctuations) both in the AP and ML directions (Duarte and Zatsiorsky, 2001; Blázquez et al., 2009). The scaling exponent (DFA-α) computed under conditions of quiet standing has been used to express differences in postural control strategies of younger and older adults (Duarte and Sernad, 2008), healthy individuals and individuals with health conditions (Moretto et al., 2021), overweight and non-overweight children and adolescents (Wiesinger et al., 2022), and between individuals with different motor skill levels (Ko et al., 2018). Several studies that applied DFA on CoP time series during quiet standing in healthy adults and in individuals with a health condition revealed two (Blásquez et al., 2009; Minamisawa et al., 2009) or three scaling regions (Kuznetsov et al., 2013) in their participants’ respective diffusion plots. That is, clearly distinct slopes for different ranges of window sizes (or time scales) with determinable transition points between them. This means that there can be different degrees and types of diffusiveness in postural patterns depending on the time scale under consideration, with results of previous work implicating persistent dynamics in short-term scaling regions and anti-persistent dynamics at long-term scaling regions (Collins and De Luca, 1993; Blásquez et al., 2009).

The two (or more) scaling regions observed in diffusion plots of CoP time series have been given different functional interpretations. For instance, it has been suggested that the dynamics captured in each scaling region reflect engagement of different types of control loop: open-loop control at short-term scaling region—resulting in drift—and closed-loop control over long-term scaling region to reign in the body and keep the CoP close to an equilibrium point (Collins and De Luca, 1993; Zatsiorsky and Duarte, 1999). Also, Riccio (1993) and Riley et al. (1997) have proposed that the persistent diffusion process at short-term scaling region serves an exploratory (perceptual) function, guiding adjustments at long-term scaling region whose signature is anti-persistence. Milton (2013) suggested that the transition from persistent to anti-persistent dynamics during quiet standing is a signature of intermittency. He specifically proposed a “drift and act” hypothesis that suggests control processes kick in only when CoP displacements exceed a threshold. Specifically, at short-term scaling region when deviation of CoP from an equilibrium point is small, there is no correction, resulting in drift, consistent with persistent dynamics. When CoP gets close to the margin, sensorimotor processes kick in to reign it back, resulting in anti-persistent dynamics at long-term scaling region.

Intermittency has recently been conceptualized more generally as the capacity of a system to switch between multiple modes of behavior, implicating engagement of different processes (some more proactive or open-loop like and others more reactive or closed-loop like). The different processes are additionally related to different functions, for example, responding to immediate task demands or exploration of context beyond the confines of ongoing task dynamics for events that unfold at slower or finer time scale (see Kelty-Stephen et al., 2021; Mangalam and Kelty-Stephan, 2021). Intermittency is thus an expression of flexibility of the postural control system that serves ongoing task demands but at the same time “respects a degree of looseness” from those demands to “check in with the broader context” just beyond immediate demands (Kelty-Stephen et al., 2021). One goal of the present study was to examine whether DFA can reveal postural adjustments that reflect both the tendency of the postural system to respond to task demands—beyond balance—under a gradually changing context.

**FIGURE 1:**
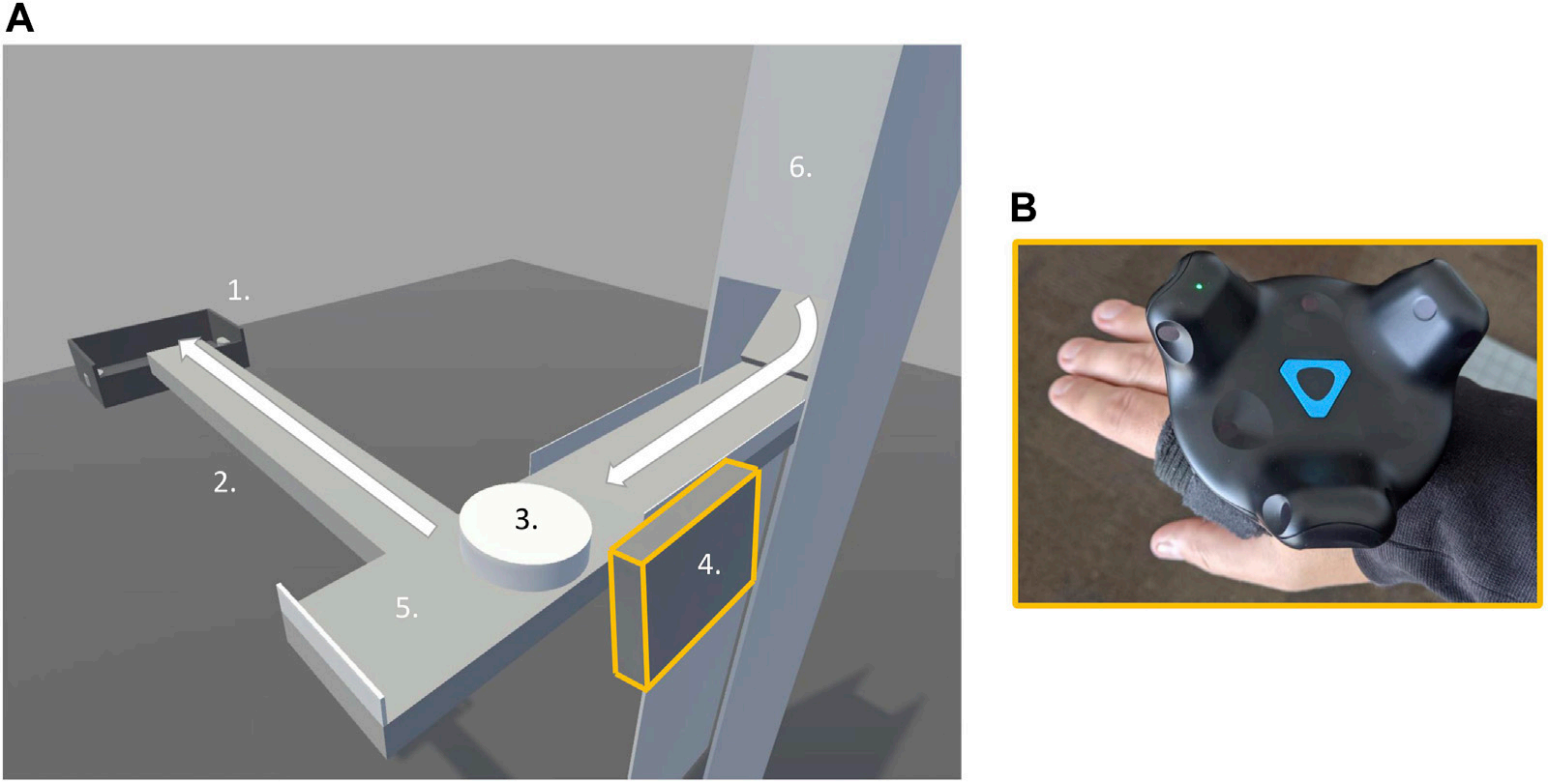
Transportation task in virtual reality. **(A)** shows the virtual reality environment, 1. Goal container, 2. Bridge, 3. Puck, 4. Virtual pad, 5. Starting area, 6. Dispenser. **(B)** shows the device attached to participant’s hand used to control the virtual pad.

To this end, we asked participants to stand on a force platform while performing a transportation task in virtual reality (VR) in which they had to move virtual objects (“pucks”) sequentially to a container positioned at the end of a narrow surface extending in front of them–similarly to working on an assembly line (see Figure 1 for an overview of the task setup). This task *demanded* recurrent upper limb movements primarily in the sagittal plane for successfully transporting each of the 65 virtual objects. In this paradigm, we also introduced a slow change (relative to the change in arm position overtime) in the broader conditions surrounding task performance. Specifically, virtual objects were delivered sequentially, and the interval between them was gradually decreased after every five pucks. Importantly, participants were free to choose how to transport the virtual object; that is, they could choose to push them all the way to the container (implicating large amplitude of arm movements) or hit them in the direction of the container at any point of the narrow surface (using relatively smaller amplitudes of arm movement). Our previous work shows that (most) healthy young adults transition from a push to a hit strategy (either gradually or abruptly) as a function of the rate at which objects appear on the scene (Nordbeck, 2020). We take this behavior as evidence that individuals, while responding to the demand of moving the arm to transport the virtual objects, remain “open” to explore and respond to changes in task context.

Prior work showed that the dynamics of postural adjustments to preserve balance (in particular the degree of diffusion at short-term scaling region) trends toward the temporal structure of movements imposed on the body through translations of the surface of support on that same direction (Rand et al., 2015; Rand and Mukherjee, 2018). Thus, we assumed that responsiveness of the postural system to task demands would be reflected in the degree of entrainment of postural dynamics to the rhythmic structure of the arm movements in the sagittal plane required for successful transportation of virtual objects to the container. The upper limb task in our study could be considered discrete (Hogan and Sternad, 2007) because participants performed one arm movement per puck with a pause before initiating the next movement. Similar discrete upper limb tasks, when performed repetitively over a period of time, lead to the development of rhythmicity in the movement (Zhang and Sternad, 2019). If the postural system is “locked” into task constraints, we should see strongly persistent dynamics in CoP signal (DFA-α > 1.5) at the short-term scaling region and an anti-persistent dynamic (DFA-α < 0.5) at the long-term scaling region; a pattern observed when postural patterns are structured by rhythmic motions (Rand et al., 2015; Rand and Mukherjee, 2018). However, if the postural system is responsive to task demands but “respects a degree of looseness” from those demands to “check in with the broader context” (Kelty-Stephen et al., 2021), a different pattern would be expected at the long-term scaling region for the ML direction as it is less directly structured by the arm movements in the sagittal plane. For the ML direction, rather than anti-persistence (supporting return to an equilibrium point), we expect context-checking explorations characterized by a low degree of persistent dynamics with DFA-α between 0.5 and 1.

The application of DFA to physiological time-series has not been without its problems and caveats. For example, physiological time-series such as CoP time series are usually bounded (because there is a limited area within which posture can be maintained), whereas an fBm is typically unbounded (Deligniéres et al., 2005). A proposed solution to this issue—one we will apply here—is to integrate the signal before analysis, such that signals are not bounded and the fractal properties of the original time series can be inferred from the integrated one (Deligniéres, et al., 2003).

Researchers have also highlighted the need to distinguish between fGn and fBm signals prior to analysis since this affects parameter estimation (Eke et al., 2002; Deligniéres et al., 2006). However, it can be difficult to classify certain dynamics as one or the other *a priori*. Most importantly, the same signal may contain one region with one dynamic and another with a different one, as is the case with CoP time-series. The application of DFA to these cases is appropriate, however, since the analysis is insensitive to the fGn/fBm dichotomy, as long as a sufficient number of series are considered (Deligniéres et al. (2005). When signal classification is not possible, one should avoid calculating the Hurst exponent from DFA-α and directly use the latter to interpret the phenomenon under consideration. In this study, to interpret the nature of postural dynamics at relatively short- and long- term scaling regions as persistent, anti-persistent, or random, we will employ the cut-offs based on DFA-α highlighted above.

One other issue to consider comes from a recent study by Carpena et al. (2021). They found that in simulated data with a known slope there are computational biases at short term scaling regions such that the slope is overestimated. In their study the problematic region spans 3 to 16 samples, but for a physiological measure using a rate of 100 Hz—such as CoP time series—the region would correspond to a range between 30–160 millisecond. A strategy to circumvent this issue—one we employed here—is to set the minimum scale in the DFA to 160 ms (i.e., 2^4 samples). The results in Carpena et al. (2021) may, therefore, not have a large impact on high-rate (“continuous”) physiological time-series, but be more relevant to “event-based” time-series such as heartbeats or stride times. For these kinds of signals, it is more difficult to remove short-term scaling regions since 4–16 events may be too substantial to disregard.

When using DFA on signals expected to have two scaling regions, which is the case here, there are a few issues that the reader should be aware of. Bryce and Sprague (2012) showed anomalies at the very short time scale of < 8 samples, where the slope was double the value of the actual (theoretical) *H* (for an fBm signal, *H* = 0.3 signal). They also showed that the estimated *H* was smaller than the true (theoretical) *H* up to approximately 50 samples (for an fGn signal, *H* = 0.5 signal).

Lastly, Bryce and Sprague (2012) showed that there may be artifacts due to non-linear trends in the data since DFA has been shown to not in fact detrend data. However, Kantelhardt et al. (2001) demonstrated that DFA is more sensitive to non-linear, slowly oscillating trends compared to faster ones, which disturb the scaling behavior much less.

As the prior discussion suggests, seemingly subtle methodological choices can affect accuracy and precision of DFA-α estimates (Deligniéres and Marmelat, 2013). For example, the choice of spacing (step size) between the window sizes at which the fluctuation function is evaluated has been shown to affect the estimates of the alpha scaling exponent and the variability of the estimate (Almurad and Delignières, 2016; Liddy and Haddad, 2018). Originally, all possible window sizes up to half the length of the signal were used in DFA analysis. However, log-transformation performed to get the diffusion plot leads to a greater density of points at larger window sizes (see Figure 1 in Almurad and Delignieres, 2016). The fluctuation function values at longer scales are also less robust because of the smaller number of windows used for their calculation. As a result, there is greater uncertainty in determining the slope of the long-term region of the diffusion plot. Yet it receives more emphasis for DFA-α estimation because the linear regression fit assumes similar weight for each point on the diffusion plot. Almurad and Delignières (2016) showed that even-spacing of the scales in the log-space (see their Figure 1, right panel) produces less variable estimates of the DFA-α. However, this still leaves the question of what step size is optimal for physiological and behavioral data. Therefore, a second goal of this study is to compare evenly-spaced DFA with two step sizes, 0.5 and 1.0 in log_2_ units, applied to a CoP time series while a person performs a functional upper limb task. We chose these step sizes because step size of 1.0 is commonly used in the DFA analysis (Gao et al., 2006); however smaller step sizes, such as 0.5, increase the number of points in the diffusion plot and could provide better resolution for identifying linear scaling relations in the log-log plot (Riley et al., 2012).

We examined if DFA step size (0.5 vs. 1.0 in log_2_ units) matters for estimating DFA-α and crossover points between scaling regions. Moreover, we examined if step size changes the pattern of results obtained from analyses directed at testing the hypotheses. With respect to the effect of step size, we hypothesized that estimating DFA-α using two evenly-spaced step sizes (0.5 and 1.0 in log_2_ units) would produce similar estimates of the short- and long-term scaling regions but different estimates for the crossover point between scaling regions.

## 2 Materials and methods

### 2.1 Experimental setup and measurements

#### 2.1.1 Participants

Forty-four undergraduate students from the University of Cincinnati participated in the study in exchange for course credit (*M*_*AGE*_ = 18.84 ± 1.38 years). Forty participants self-reported being right-handed and four left-handed. All participants had normal or corrected-to-normal vision, and none had recent injuries, neurological, motor, or balance disorders affecting their movements. The University of Cincinnati Institutional Review Board approved all study procedures and methods.

#### 2.1.2 Materials and apparatus

Participants wore a VIVE virtual reality (VR) headset (HTC Corporation, Bellevue WA) that displayed a virtual environment (described below) created in Unity (v.2019; Unity Technologies, San Francisco, CA). The virtual environment was rendered at a rate of 90 Hz. CoP in two directions, ML and AP, were measured continuously using a force platform (AMTI AccuSway+, Advanced Mechanical Technology, Watertown, MA) and Balance Clinic software at a sample rate of 100 Hz.

#### 2.1.3 Procedure

Participants stood on the force platform in a comfortable stance with feet positioned approximately shoulder-width apart. Within the VR environment, participants viewed a narrow table at a height of 1.01 m directly in front of them, a goal container positioned 0.91 m ahead of them, and a narrow bridge connecting the two (see Figure 1A). At the right end of the narrow table, a puck dispenser was placed so that 65 pucks could be presented to participants one by one. Participants were instructed to use a virtual pad—experienced as an attachment to the hand—to move as many pucks as possible from the narrow table across the bridge and into the goal container (see Figure 1B).

Importantly, participants completed the task under gradually increasing contextual demands. In particular, the presentation rate of the pucks was altered throughout performance, beginning with an interval of 7 seconds. Then, after every five pucks, the presentation rate changed from seven (slowest) to one second intervals (fastest), at 0.5 s increments (resulting in 13 intervals total). To be successful in the task and minimize performance losses when the time interval between pucks got shorter and shorter, participants had to remain “open” to explore movement strategies that corresponded to the increasing contextual demands. The time pressure that was part of the task “pushed” participants into a rhythmic arm movement pattern (Sternad et al., 2013). The task lasted 260 s, and CoP was recorded in two directions (ML and AP), resulting in two time series of lengths *N* = 26,000 per participant.

### 2.2 Analytical setup and output variables

#### 2.2.1 Detrended fluctuation analysis

The first step in the DFA is to integrate the CoP time series, which makes the assumption that the original CoP position time series is an instance of a stochastic fGn process—a stationary process with correlation properties characterized by *H* = DFA slope (Deligniéres et al., 2011). While CoP is likely non-stationary (e.g., the mean or variance is time-dependent) when recorded for short durations (Riley et al., 1999), longer trials of standing must show bounded, stationary dynamics because CoP fluctuations must remain within the base of support by definition to avoid a fall. In this sense, approximating long-term CoP as stationary fGn-like time series appears reasonable, however, it is clearly a simplification of the physiologic processes underlying CoP dynamics (Kuznetsov et al., 2013).

MATLAB (MathWorks, Natick, MA) functions implementing evenly-spaced DFA were adapted from the Nonlinear Methods for Psychological Science APA ATI training and functions provided by Jianbo Gao (http://www.gao.ece.ufl.edu/). The even-spacing method is outlined in Almurad and Delignières (2016; Section 2.3). Each data series was analyzed with DFA twice, once with step size set to 1.0 and then 0.5. The minimum window size was set to 2^3^ and maximum to 2^12^, and the number of calculated window sizes was 9 for step size 1.0 and 19 for step size 0.5. The log-log scale values (alpha and window size) were retained for the Two-Region Fit (TRF) analysis (see below), and the slope of the log-log values was calculated, resulting in four calculated slopes per participant (one calculated slope per movement direction [ML and AP] and step size [0.5 and 1.0]).

According to Carpena et al. (2021), there is a short time-scale region where spurious scaling occurs when analyzing a data-series with a continuous DFA. Therefore, we simulated data for the most affected signal in their study (fGn, *H* = 0.2) and replicated their result, namely, that there is a clear, small region at short time-scales that overestimates the true *H*. We then ran the continuous DFA on our observed data-series but could not replicate the spurious scaling at 4–16 samples. However, the evenly-spaced DFA with 0.5 and 1.0 step size shows a small region with the overestimation for the observed data from 4 to 8 samples. Along with Bryce and Sprague’s (2012) findings of inaccurate estimates at very short time scales, we set the minimum DFA scale to 8 samples (2^3^ window size).

#### 2.2.2 Two-region fit

The procedure for the TRF was adapted from Kuznetsov et al. (2013). Working from the diffusion plot, one must determine whether linear scaling is present and whether it is present in one or more regions of the plot. We first visually inspected every individual DFA plot of the CoP data to evaluate the presence of fractal scaling and the number of possible linear scaling regions in these data. Visual inspection suggested that in all cases there were two scaling regions: one at relatively shorter and another at relatively longer time scales. We fitted the following piecewise linear model with two regions and one crossover point at log_2_(Fn) = k between them to the DFA curves,

$$y=b_{1}x+a_{1}\;for\;x\;<\;k,$$

$$y=b_{2}x+a_{2}\;for\;x\;>\;k,$$

In order to choose the best two-region models, we evaluated the global goodness of fit for all two-region fits and all possible breakpoint locations between log_2_ w values from 5% to 80% of all log_2_ w. The reason for these particular cut-off points was that the visible boundaries of the regions were typically within this range of window sizes. We allowed the fit to start at either the short or the long scaling region and allowed up to one log_2_ w separations between the regions. This was done so that the fits were not constrained to continuous linear regions only. Goodness of fit was quantified as the residual sum of squares (RSS),

$$RSS=\Sigma {\left(y_{i}-y\right)}^{2}$$

After iteratively fitting the defined 2-region models, we found the 10 best-fitting models and chose the one with the longest first region. We prioritized the identification of the first scaling region because it seemed to be the most reliable, clearly defined, and longest scaling region based on the preliminary visual inspection of all time series.

### 2.3 Statistical analysis

Statistical analysis was performed using custom RStudio (RStudio, PBC, Boston, MA) scripts. Prior to statistical analysis, data were subjected to outlier analysis (lower bound: Q1 - IQR*2.5; upper bound: Q3 + IQR*2.5). Five outliers were excluded from the short-term scaling region analysis, and three from crossover point analysis. The DFA-α for the short- and long-term scaling regions and the crossover point stratified by movement direction (AP and ML) and step size (0.5 and 1.0) are reported as mean and confidence intervals in Table 1.

**TABLE 1:** Mean crossover points and DFA estimates (confidence interval in brackets) by step size and movement direction.

Step size and movement direction	Crossover point	DFA-α for short-term scaling region	DFA-α for long-term scaling region
0.5 and AP	7.93 [7.82, 8.04]	1.85 [1.84, 1.87]	0.52 [0.48, 0.56]
0.5 and ML	7.92 [7.82, 8.02]	1.81 [1.80, 1.83]	0.66 [0.62, 0.71]
1.0 and AP	8.48 [8.35, 8.61]	1.79 [1.78, 1.81]	0.53 [0.49, 0.58]
1.0 and ML	8.37 [8.24, 8.49]	1.76 [1.75, 1.78]	0.68 [0.62, 0.73]

Note. DFA, detrended fluctuation analysis; ML, medial-lateral; AP, anterior-posterior.

Mixed effects models were used to determine whether there were effects of movement direction (to address our first goal), and step size (to address our second goal), and their interaction on the DFA-α of the two scaling regions (short- and long-term scaling regions) and crossover points. We did not run a model on the overall DFA because the diffusion plots and TRF analysis indicated the presence of two distinct scaling regions. Step size and movement direction were entered as fixed effects, and participant as a random effect. A backward stepwise approach was used for model building as described in West et al. (2015). Models were trimmed by removing nonsignificant effects individually, progressing from higher-to lower-order interactions. At each step, we compared the deviance (−2 Log Likelihood; −2LL) between a larger model and a simpler nested model that excluded the predictor under analysis. The change in −2LL follows a chi-square distribution with degrees of freedom equal to the difference in the number of parameters between nested models, allowing for a test of statistical significance. The final model included only higher-order interactions that significantly improved model fit (and all component lower-order interactions and main effects). Level of significance for all tests was set at *p* < 0.05. Only significant effects will be reported.

## 3 Results

### 3.1 Overall description of DFA-alpha and crossover point

**FIGURE 2:**
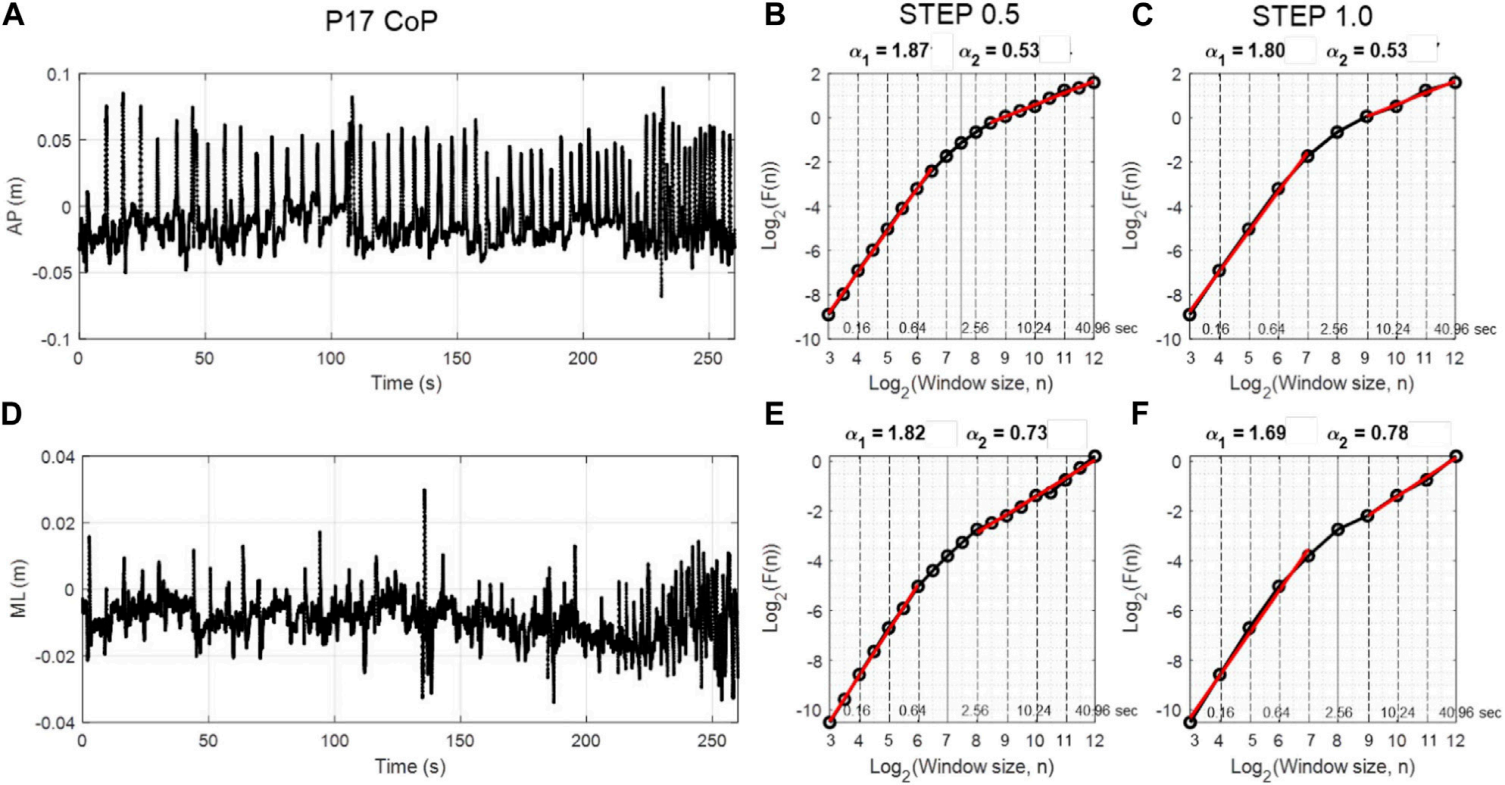
Panels **(A,D)** show sample data from AP and ML CoP time series from a single participant (P17) as they performed the puck transportation task. The DFA plots using log2 step size 0.5 and 1 are presented for the same CoP data in panels **(B,C)** and **(E,F)**, respectively. Two scaling regions (α1 and α2) were evident in all DFA plots regardless of the step size and CoP direction. The window sizes at which the DFA fluctuation function was estimated are presented in log2 units below the axis and in seconds above the axis.

Figure 2 depicts an example AP and ML CoP time series and diffusion plots from a single participant using DFA step size 0.5 and 1.0. As expected (and important for further analysis), DFA diffusion plots revealed two scaling regions in the AP and ML CoP time series (Figures 2B, C, E, F). In the AP direction using the DFA step size 0.5, the first scaling region was present for the shorter time scale ranging from window size 3 to 6.5, which corresponds to 80 ms to 0.90 s. To convert log_2_ window size into seconds, raise 2 to the window size value and divide by 100 Hz; e.g., window size 6.5 corresponds to 2^6.5^/100 = 0.90. The second scaling region was present for the longer term scale (3.62–40.96 s). The crossover point was identified at a window size of 7.5 (or 1.81 s). The DFA plot using step size of 1.0 produced a higher estimate of the crossover point (8), slightly lower alpha estimate for the short-term region and similar alpha estimate for long-term region.

**FIGURE 3:**
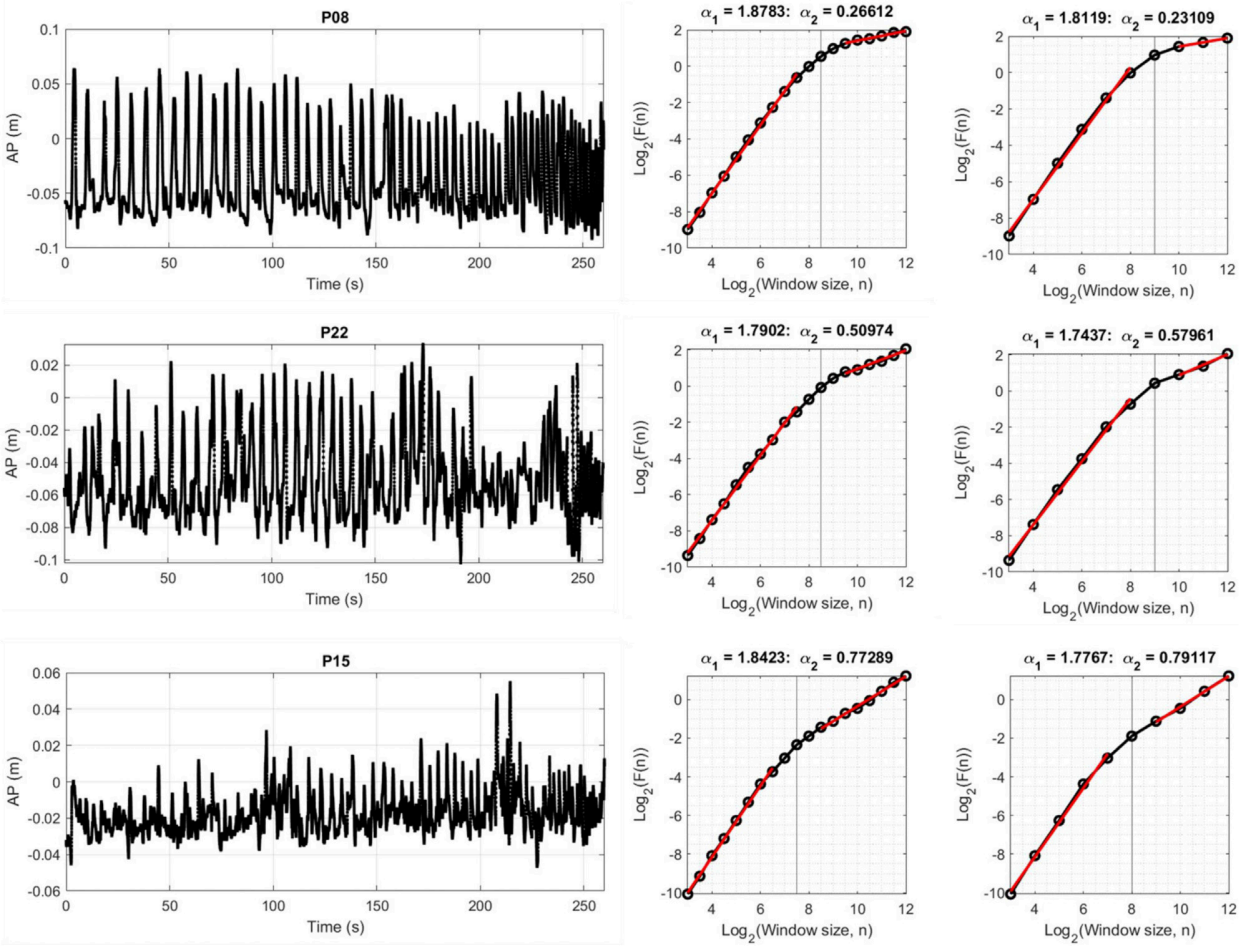
Three representative subjects illustrating AP CoP and corresponding DFA plots for step size 0.5 and 1.0. The first region generally had α values around 1.8, while the second region has trials with α less than 0.5, around 0.5, and greater than 0.5. The α for the first scaling region was generally slightly higher when using 0.5 step size compared to 1.0. The same pattern held for the ML direction.

Overall description of average DFA-α in each region and average location of the crossover points for both movement directions and step sizes are reported in Table 1. As expected, the short-term scaling region showed DFA-αs in the hyper-diffusive range, regardless of step size. With 0.5 step size, hyper-diffusiveness was slightly stronger (AP *α* = 1.85; ML *α* = 1.81) compared to 1.0 step size (AP *α* = 1.79; ML *α* = 1.76). For the longer time scale, DFA-α estimates suggest persistent dynamics. DFA-α values for the AP were closer to random dynamics for both 0.5 and 1.0 step sizes (0.5 step size, *α* = 0.52; 1.0 step size, *α* = 0.53) compared to the ML (0.5 step size, *α* = 0.66; and 1.0 step size, *α* = 0.68). The mean crossover points for step size 0.5 was 7.92 for ML and 7.93 for AP. For step size 1.0, these values increased to 8.37 for ML and 8.48 for AP. The crossover point is thus estimated at a shorter time scale when using the 0.5 log-unit step size. Figure 3 shows representative time series and DFA plots for the AP direction using two DFA step sizes.

### 3.2 Effect of step size on parameter estimation of the short-term scaling region

**FIGURE 4:**
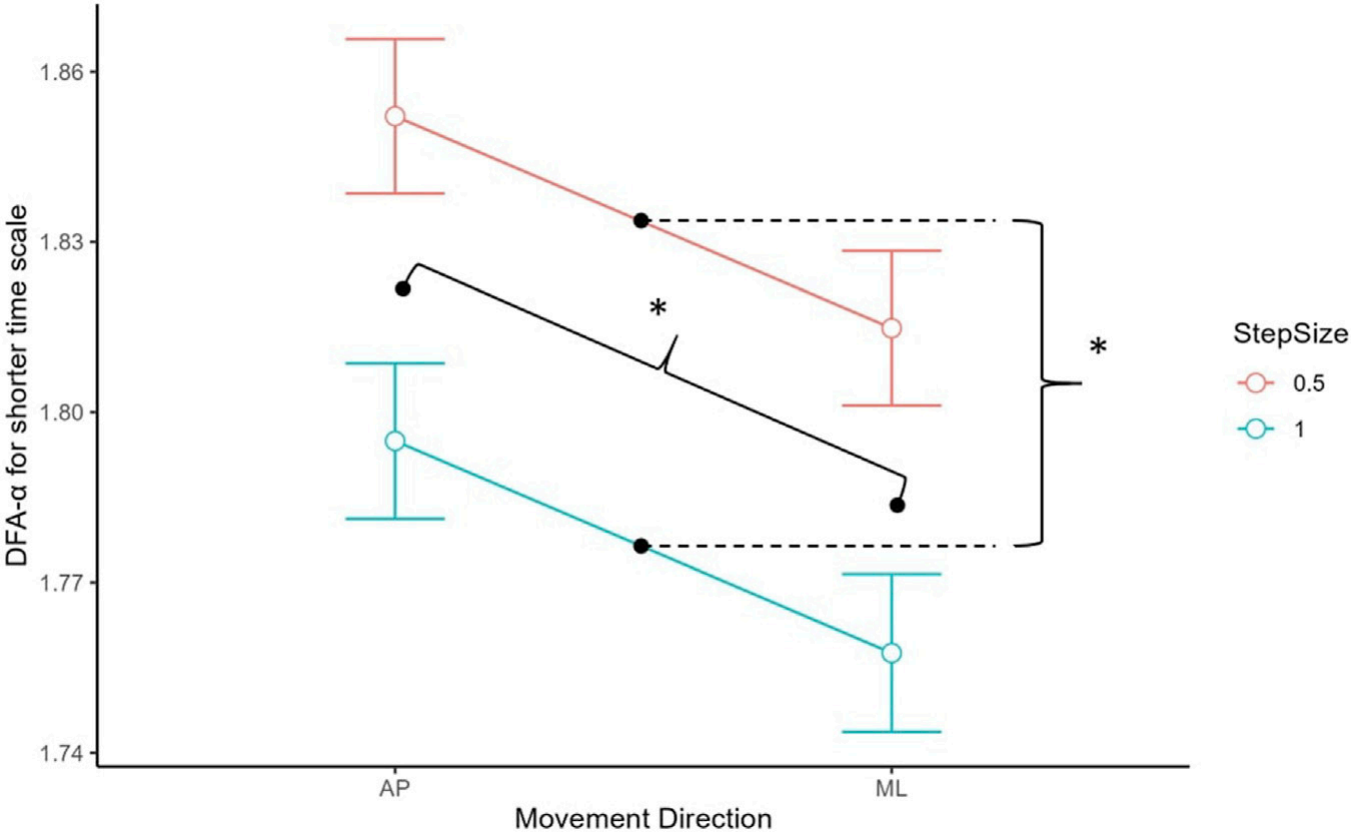
DFA-α estimate for short-term scaling region by movement direction. AP had a higher DFA-α than ML, and step size 0.5 had a higher DFA-α than 1.0. Asterisks denote statistical significance. Error bars represent 95% confidence intervals.

For the short-term scaling region, there were significant main effects of step size, *t* (123.48) = −9.96, *p* < 0.001, and movement direction, *t* (122.45) = −6.52, *p* < 0.001 (see Figure 4). The DFA-α was significantly higher when step size was 0.5 (*M* = 1.83, *SD* = 0.05) than when step size was 1.0 (*M* = 1.78, *SD* = 0.05), and in the AP direction (*M* = 1.83, *SD* = 0.04) in comparison to the ML direction (*M* = 1.79, *SD* = 0.06).

### 3.3 Effect of step size on parameter estimation of the long-term scaling region

**FIGURE 5:**
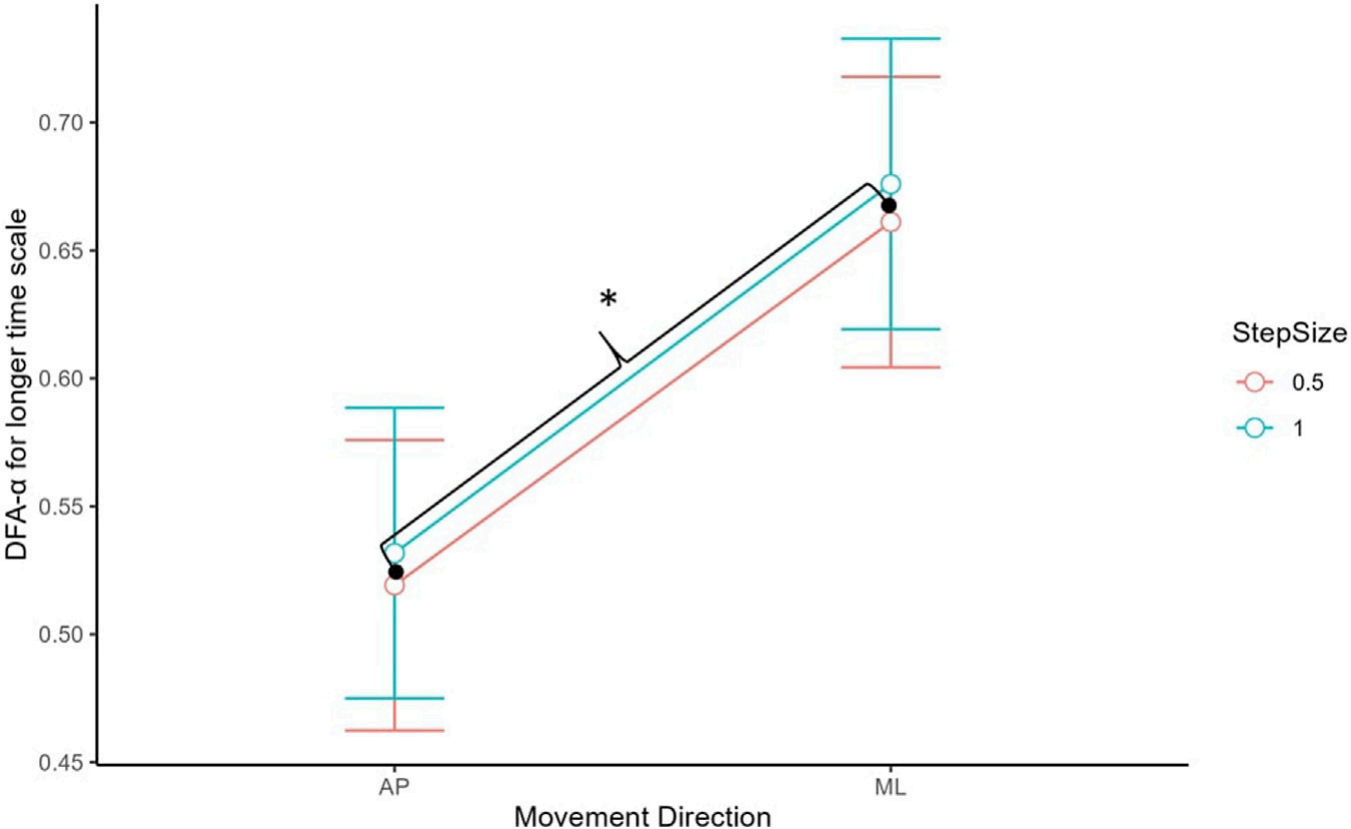
DFA-α estimate for long-term scaling region by movement direction. AP had a lower DFA-α than ML. Asterisks denote statistical significance. Error bars represent 95% confidence intervals.

For the long-term scaling region, there was a significant main effect of CoP direction, *t* (132) = 7.35, *p* < 0.001 (see Figure 5). The DFA-α was significantly higher for the ML (*M* = 0.67, *SD* = 0.20) than AP (*M* = 0.53, *SD* = 0.17) direction.

### 3.4 Crossover point

**FIGURE 6:**
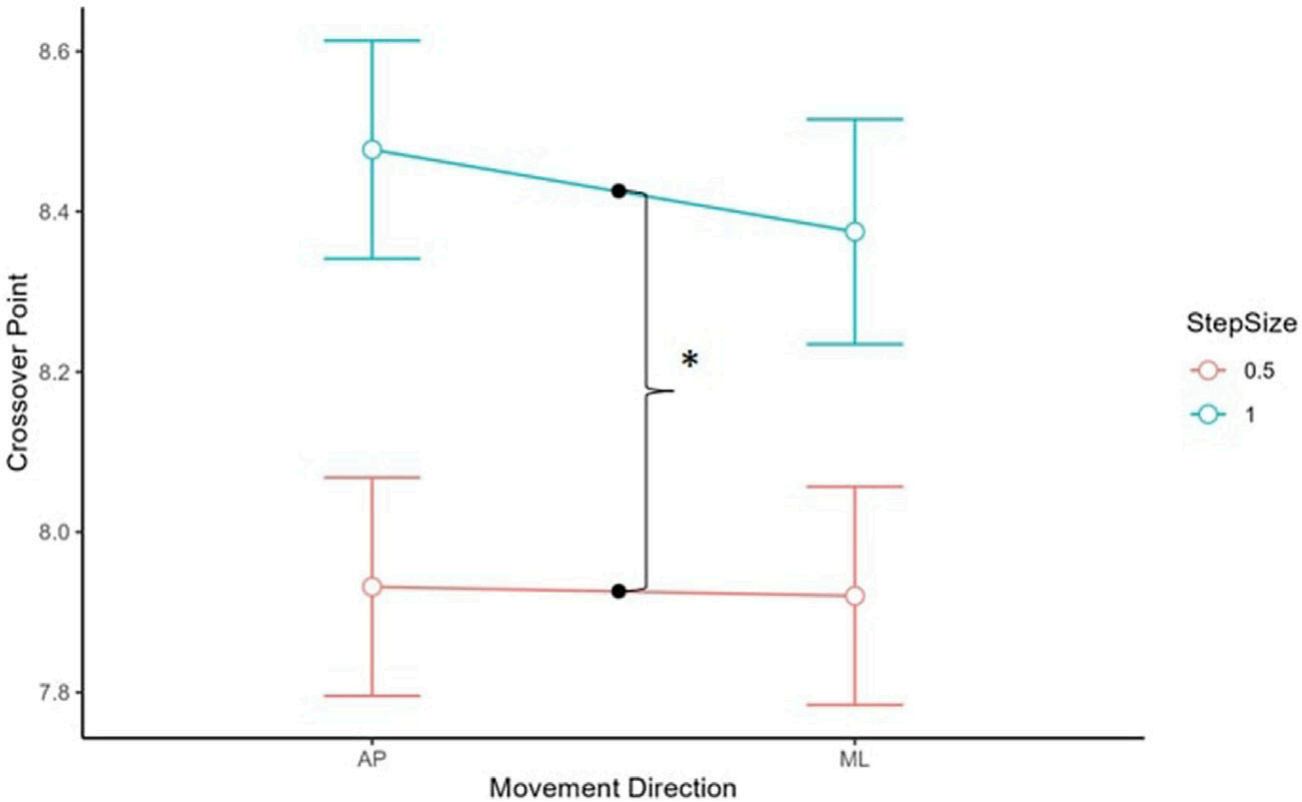
Crossover point estimates for AP and ML for 0.5 and 1.0 step size. A step size of 0.5 generated lower CoP estimations than 1.0. Asterisks denote statistical significance. Error bars represent 95% confidence intervals.

For the crossover point, there was a main effect of step size, *t* (129.47) = 8.61, *p* < 0.001, such that the crossover points were generally higher with the log_2_ unit step size of 1.0 (*M* = 8.42, *SD* = 0.50) compared to 0.5 (*M* = 7.93, *SD* = 0.41) (see Figure 6).

## 4 Discussion

Our study evaluated CoP displacement in the AP and ML directions presented by healthy individuals when performing a functional upper limb task in virtual reality. Our findings partially supported our hypotheses. As expected, we identified two scaling regions in the DFA plots of these CoP signals: a short-term scaling region (spanning from 80 ms to about 2.89 s) and a long-term scaling region (spanning from 2.90 to 40.96 s). In the short-term scaling region, CoP signals for both AP and ML showed strongly persistent dynamics (DFA-α > 1.5). However, at the long-term scaling region, rather than anti-persistent dynamics, CoP for AP continued indicating persistent dynamics on average (0.5 < DFA-α < 1). For the ML direction, CoP displacement also indicated persistent dynamics in the long-term scaling region and this result was in line with our hypothesis. In this study, we also evaluated if a subtle methodological choice—the use of two different, evenly-spaced step sizes in the DFA analysis (0.5 and 1.0 in log_2_ units)—could affect the estimates of DFA-α and crossover point. Again, our findings partially supported our hypotheses. As expected, the two step sizes produced different estimates for the crossover points (with smaller estimates for the 0.5 step size). However, although the two step sizes produced similar estimates of DFA-α for the long-term scaling region, it did not for the short-term scaling region. Below, we discuss the functional interpretation of our findings and potential implications.

### 4.1 Postural dynamics supporting task performance

Consistent with prior work investigating the dynamics of postural adjustments to preserve balance (Riccio, 1993; Riley et al., 1997; Milton, 2013), we also found a persistent diffusion process at short-time scales. This persistent behavior suggests that, during the performance of the upper limb task in VR, participants’ CoP deviated from an average equilibrium point toward the edge of the base of support and did not tend to “correct” itself in the short term. Riccio (1993) argued that this persistent dynamic serves an exploratory (functional) purpose, and as long as it does not threaten balance, there is no need to switch to an anti-persistent dynamic (Milton, 2013). In our dataset, short-term scaling region slopes were between 1.68 and 1.93, which is consistent with a hyperdiffusive fBm process. Such dynamics would be consistent with the relatively rhythmic CoP fluctuations observed during the object transportation task (see Figure 1). These rhythmic fluctuations were related to the forward-backward movement of the body as participants reached forward to deliver the puck.

At the long-term scaling region, we expected to see an anti-persistent dynamics in the AP direction, that is, a tendency to return the position of the CoP to an equilibrium point to ensure balance. However, DFA-αs indicate remaining in the persistent range (DFA-α ∼ 0.53). Despite this finding, it is worth noting that the values of DFA-α for the long-term scaling region dropped considerably in comparison to the short-term scaling region (from ∼ 1.83 to 0.53). That is, the CoP dynamics at the AP direction became less persistent at long-term scaling regions. The value at this scale likely reflects the overall drifting of the instant equilibrium point around which participants centered their balance (Zatsiorsky and Duarte, 1999). A simple heuristic simulation showed that adding a weakly diffusive dynamic (fBm *H* ∼ 0.3) to a sinusoid reproduced the pattern of scaling exponents of the long-term scaling region observed in our study. When the drift is weak, the long-term scaling region slope is close to 0, but becomes closer to 0.5 when the diffusion is made stronger.

Our task has an interesting characteristic that may help us understand why we did not see a transition between persistent to anti-persistent dynamics at different time scales. The presentation rate of the virtual objects varied constantly (every five pucks, the delivery interval between one puck and another decreased by 0.5 s), requiring that participants maintained a certain “degree of looseness” and flexibility in their postural control throughout the task. Together with the finding that AP DFA-αs were less persistent compared to ML, these results may indicate that participants needed to maintain some exploratory movement also in the AP direction. The expectation that movement in the ML direction would result in low degree of statistical persistence at long-term scales (DFA-α ∼ .67) is consistent with Kelty-Stephen et al. (2021), in that, this movement direction should be less structured by arm movement, which occurred mostly in the sagittal plane. Finding low values of CoP persistence at long-term scaling regions for both AP and ML may simply indicate that participants needed to remain “open” to explore movement strategies in both movement directions (albeit to a lesser degree for AP) as a demand from the continuously changing context. Such dynamics are also consistent with the generally bounded nature of the CoP time series during this task, in which people need to enact some postural corrections to remain in generally the same location over a longer period of time.

### 4.2 The step size choice

Although the DFA estimates and interpretation can be affected by methodological choices, the effects of step choice on DFA-α and crossover between scaling regions have never been assessed. While several studies used DFA to study postural control in humans (Blázquez et al., 2010; Duarte and Stenard, 2008; Saraiva et al., 2023 as a few examples), to our knowledge none have explicitly reported the choice of spacing (step size) between window sizes. For our dataset, decreasing step size from 1.0 to 0.5 resulted in a higher DFA-α estimate. Since all DFA-αs were in the stronger region of the hyper-diffusive range, this difference was only quantitative and did not alter the class of observed dynamic (e.g., from hyper-diffusive to underdiffusive). Although the difference in step size did not result in qualitative differences in our study, it is still important to consider since other tasks or kinds of signals might generate DFA-α estimates closer to the borders of qualitative change. For such datasets, our analysis shows that step size may influence the estimation of DFA-α to the point of finding qualitative differences.

With respect to crossover point, we found that using a smaller step size of 0.5 log_2_ units led to a lower estimate of the crossover point time (2.43 s) as compared to step size of 1.0 (3.43). Given that previous studies indicated that the crossover point is around 1s (Collins and De Luca, 1993), we recommend that smaller step size is used as it appears to provide a more accurate estimate. Another downside with using the large step size of 1.0 log_2_ units is that some of the long-term region DFA-α were based on only three fluctuation function estimates, which may lead to more variable DFA-α estimates compared to step size of 0.5 log_2_ units. Furthermore, one of the more subtle (but equally problematic) aspects of only using a larger step size is that one might miss the spurious or otherwise anomalous estimates at short range. One might therefore (even unbeknownst) introduce inaccurate results in the literature instead of, for example, excluding the problematic region by adjusting the minimum window size from which to estimate the parameters. We, therefore, recommend in the general case to use a step size of 0.5 to improve accuracy and guide other methodological decisions in one’s analysis.

### 4.3 Limitations and future directions

Our DFA analysis focused on comparing only two step size values. We performed the DFA analysis using a step size of 1.0 because this value is commonly used (Gao et al., 2006) and our rationale for using a step size of 0.5 was to increase the number of points in the diffusion plot, and consequently, increase the resolution for identifying linear scaling relations in the log-log plot. However, it is possible that step sizes smaller than 0.5 would provide even better resolution and new useful information. Future studies should investigate the effect of not only two but a continuous range of step sizes on DFA estimates. Also, while it is important to assess parameters on real (human movement generated) data, it would also be advantageous to combine this with analyzing synthetic data with a known DFA-α (similarly to Almurad and Delignières, 2016). That way, deviations from the known DFA-α could be assessed in a manner that is easier to interpret compared to when the known DFA-α is unavailable. Furthermore, we have applied DFA analysis to relatively rhythmic CoP time series—due to the influence of the rhythmic movement of the upper limb during the puck transportation task. Therefore, our results may not be generalized to the many other types of physiological and behavioral time series that DFA has been applied to. Future studies should explore the impact of methodological choices on other signals such as heart rate variability, electroencephalogram signals, gait patterns, and non-rhythmic patterns alike.

To conclude, our analysis showed that DFA was able to reveal the postural adjustments supporting performance of the proposed upper limb task under variable demands in both AP and ML directions. Our analyses also showed that methodological choices in the DFA analysis can have effects on the estimation of both DFA-α and crossover points. In our task, those differences were quantitative (i.e., remained within one category of dynamics such as the hyper-diffusive range for shorter time scales) but for other tasks, movements, or signals, the estimated range may cross a qualitative boundary (i.e., hyper-diffusive, diffusive, or underdiffusive), and have even larger interpretational consequences. We, therefore, recommend transparency when reporting the methodological choices of the analysis so that inadvertent false comparisons can be avoided, and different studies can be appropriately compared. The results of the CoP dataset used here suggest that using step size of 0.5 log_2_ units is preferable to 1.0 when performing evenly-spaced DFA in data with similar characteristics as ours.

## Data Availability

The raw data supporting the conclusion of this article will be made available by the authors, without undue reservation.
